# Child Adjustment During COVID-19: The Role of Economic Hardship, Caregiver Stress, and Pandemic Play

**DOI:** 10.3389/fpsyg.2021.716651

**Published:** 2021-08-18

**Authors:** Rachel B. Thibodeau-Nielsen, Francisco Palermo, Rachel E. White, Alaina Wilson, Shannon Dier

**Affiliations:** ^1^Department of Human Development and Family Science, University of Missouri, Columbia, MO, United States; ^2^Department of Psychology, Hamilton College, Clinton, NY, United States

**Keywords:** COVID-19, child adjustment, pretend play, self-regulation, caregiver stress

## Abstract

The coronavirus disease 2019 (COVID-19) led to many lifestyle changes and economic hardships for families with young children. Previous research on risk and resilience highlights that children's adjustment to family hardships is influenced by caregiver stress, but individual child behaviors and characteristics may protect children from negative outcomes. Interestingly, many children have been reported to incorporate COVID-19 themes in their pretend play. Theory suggests children may do so to cope with pandemic-related stress, but no empirical studies have explored this possibility. The purpose of this study was to understand the process by which COVID-19 economic hardships experienced by a family were related to children's emotional well-being and development and to investigate how this process may vary as a function of children's engagement in pandemic-related pretend play. Caregivers (*N* = 99; mostly high earning families) of preschoolers ages 3–6 years (51% girls, 82% White) living in the United States participated in an online survey at two time points during the pandemic. Result revealed that COVID-19 economic hardships were related to increased caregiver stress, which, in turn, was associated with children's emotional distress and poorer self-regulation. However, engaging in pandemic-related pretend play appeared to protect children's well-being by weakening the adverse association between caregivers' stress and children's emotional distress. Thus, addressing caregiver stress levels and allowing children an outlet to cope with challenges through pretend play could have crucial protective effects on early development and well-being during times of crisis.

## Introduction

“*Hotel is closed. Vacations are canceled. Everybody go home!”*

*-4-year-old engaging in pretend play during the COVID-19 pandemic*

The coronavirus disease 2019 (COVID-19) pandemic upended daily routines for millions of families. To slow the spread of COVID-19, governments limited social gatherings, leading many schools and workplaces to temporarily close. Families sheltered-in-place, hastily adjusted to school and work closures, and limited their contact with non-household individuals (Schuchat, 2020). Many families also experienced significant economic hardship (Gassman-Pines et al., 2020); over 50 million adults with children reported a loss of income from March to October of 2020, and 59 million (60% of those surveyed) experienced difficulty covering household expenses (United States Census Bureau, 2020). There is broad interest in understanding how children are coping with these pandemic-related challenges (Coller and Webber, 2020), with some speculating the importance of play as a potential protective factor for children's well-being and development (e.g., Pelly, 2020).

### COVID-19, Stress, and Development

Research on disease outbreaks highlights the stress-inducing nature of events like the COVID-19 pandemic (e.g., Jones and Salathé, 2009). Although children are likely not immune to experiencing COVID-19 pandemic-related stress (e.g., Gassman-Pines et al., 2020; Patrick et al., 2020), family stress frameworks posit that environmental hardships are more likely to affect children's well-being indirectly through increased caregiver stress (Masarik and Conger, 2017; Prime et al., 2020). One recent cross-sectional study finds support for this theory during the COVID-19 pandemic (Spinelli et al., 2020), but more research is needed to understand the process by which pandemic-related hardships influence children's adjustment over time. Doing so may help identify potential avenues to support children and families during times of crisis.

Furthermore, we must consider how pandemic-related hardships and stress relate to important developmental skills, like self-regulation. Self-regulation encompasses the ability to manage thoughts and behaviors and supports relational, academic, and occupational success as well as physical health throughout childhood and adulthood (Blair and Razza, 2007; Moffitt et al., 2011). Importantly, the manner in which children respond to stressors, particularly long-lasting and unpredictable stressors like those elicited by a global pandemic, may have important implications for self-regulation development (Blair, 2010; Thompson, 2014). Thus, we examined the caregiver and child mental health processes (i.e., caregiver stress and child emotional distress) by which COVID-19 economic hardships may compromise children's self-regulation development.

### Play as a Protective Factor

After the World Health Organization declared COVID-19 a global pandemic, stories emerged of children incorporating the virus and related life-changes into their play (e.g., Cray, 2020; Pelly, 2020; Underwood, 2020). Children were reportedly diagnosing siblings with COVID-19 while playing “doctor,” instructing parents to put on masks in pretend restaurants, and treating COVID-19 as an evil villain (Cray, 2020; Underwood, 2020). Pretend play is ubiquitous in early childhood, emerging spontaneously and at predictable developmental periods for children around the world; this suggests that play may serve an important evolutionary function (Lillard, 2017). Developmentalists have noted that children use pretend play to make sense of their world, allowing them to face emotionally-laden scenarios in a safe context and providing opportunities to process and overcome negative emotions (e.g., Knell, 1993; Russ, 2004). Indeed, children who play out stressful experiences (e.g., hospitalized children playing with medical equipment, preschoolers reenacting caregiver separation) generally exhibit decreases in anxiety and distress (e.g., Milos and Reiss, 1982; Rae et al., 1989). Additionally, because children are in charge of their imaginary scenario, pretend play may also provide an opportunity for children to assert control over their environment (Clark, 2003). Thus, children may naturally incorporate pandemic-related themes in their pretend play as a way to cope with the stressful situations stemming from the COVID-19 pandemic.

### The Present Study

In the present study, we explored the process by which COVID-19 economic hardships predict children's emotional adjustment and self-regulation development and examined if engaging in pandemic play serves as a protective factor. We hypothesized that COVID-19 economic hardships would indirectly predict child emotional distress several months later via a positive association with caregiver stress. We further hypothesized that higher levels of child emotional distress would lead to poorer self-regulation skills among children. However, we believed these relations would depend on the extent to which children explored COVID-19 themes in their play, with pandemic play serving as a protective factor.

## Method

### Participants

Primary caregivers of children ages 3–6 living in the United States participated in this study by completing two online surveys. A total of 185 caregivers completed the initial Time 1 (T1) survey, and 171 indicated they would participate in a follow-up. Of those 171, 127 caregivers completed a follow-up survey at Time 2 (T2), ~3 months later; however, we excluded 28 participants because they did not answer questions for the same child on the first and second surveys. Thus, our final sample included responses from 99 caregivers. This final sample did not differ from those who completed the survey only at T1 on demographic or baseline variables. Respondents were mostly mothers (96%). Most of them reported having a college degree (91%), with average annual incomes between $100,000 and $110,000. The children were approximately equally distributed across genders (49 boys, 50 girls), were mostly White (82%), and, on average, 50 months of age (SD = 9.03).

### Procedures

Caregivers were recruited via advertisements on listserv posts and social media websites starting in May of 2020. The T1 survey was distributed via Qualtrics between May and July of 2020 (92% were completed within the first 2 weeks). Participants who completed the T1 survey were entered into a drawing to win one of 40 gift cards ranging in value from $25 to $200. In September of 2020, the T2 survey was distributed by email. All participants received a $20 gift card upon completing the T2 survey. A university Institutional Review Board approved this study.

### Measures

#### COVID-19 Economic Hardships

During the T1 survey, caregivers were asked “What changes in employment or income have occurred in your household due to COVID-19?” Caregivers selected all the options that applied to their household from the following list of possibilities: job loss by one adult, difficulty paying bills or buying necessities, having to work longer hours, filing for unemployment, applying for public assistance, and loss of equity in the stock market. From this, we calculated the total number of economic changes per household. Caregivers were also asked, “How much financial strain do you feel right now?” and responded on a 5-point scale ranging from “no strain” to “a lot of strain.” Number of economic changes and perceived financial strain were correlated, *r* = 0.53, *p* < 0.001. Thus, we standardized these variables to equate their scales and averaged them to create a COVID-19 economic hardships score. Higher scores indicate greater economic hardship.

#### Caregiver Stress

During the T2 survey, caregivers completed the Perceived Stress Scale (α = 0.89; Cohen et al., 1983) and an abridged version of the Parenting Stress Scale (α = 0.81; Berry and Jones, 1995). The Perceived Stress Scale included 10 items assessing a caregiver's overall feelings of worry and stress during the last couple of months (e.g., “How often have you felt nervous and stressed”). Caregivers responded on a 5-point scale from “never” to “very often.” The Parenting Stress Scale also included 10 items assessing a caregiver's stress during the last couple of months as a function of their parenting role [e.g., “Caring for my child(ren) sometimes takes more time and energy than I have to give”]. These items were rated on a 5-point scale ranging from “strongly disagree” to “strongly agree.” Scores on these measures were correlated, *r* = 0.54, *p* < 0.001. Thus, we standardized and averaged them. Higher scores indicate greater caregiver stress.

#### Child Emotional Distress

Caregivers completed two subscales from the Child Behavior Checklist Parent-Report Form for ages 1.5–5 during the T2 survey (emotional reactivity, nine items, α = 0.81; anxiety and depression, eight items, α = 0.70; Achenbach, 1999). We selected these subscales to capture children's emotional states during the COVID-19 pandemic. Items included questions about trouble adjusting to new routines, outward displays of negative emotions, and clinging behaviors. Caregivers indicated how true each item was during the last couple of months on a 3-point scale ranging from “not true” to “very true or often true.” The subscales used were correlated, *r* = 0.79, *p* < 0.001, so we standardized and averaged them. Higher scores indicate greater child emotional distress.

#### Children's Self-Regulation

Caregivers completed two self-regulation subscales of the Early Years Toolbox at both time points (cognitive self-regulation, five items, α= 0.63–0.70; behavioral self-regulation, six items, α = 0.73–0.80; Howard and Melhuish, 2017). During the T2 survey, caregivers were specifically asked to think about “the last couple of months.” Example items include, “Waits their turn in activities” and “persists with difficult tasks.” Caregivers indicated how true each item was of their child on a 5-point scale ranging from “not true” to “very true.” Given a strong correlation between the two subscales, *r* = 0.39–0.51, *p* < 0.001, subscale scores at each time point were standardized and averaged to create separate T1 and T2 self-regulation variables. Higher scores indicate greater self-regulation abilities.

#### Pandemic Play

Caregivers were asked in the T2 survey to describe any play their child had engaged in over the past few months related to the COVID-19 pandemic (see Appendix A in Supplementary Material for descriptions of this play). We also asked caregivers to indicate on a 7-point scale how often their child engaged in play related to the pandemic during the last couple of months, with choices ranging from “never” to “multiple times a day.” For comparison purposes, we grouped children into two categories: those who engaged in pandemic play infrequently (i.e., between never and less than once a week) and those who engaged in pandemic play frequently (i.e., once a week or more).

## Results

A table of bivariate correlations, means, standard deviations, and ranges for individual measures can be found in the Appendix B in Supplementary Material. The correlations among averaged variables used in our analyses are included in Table 1. Based on theory and research (Conger et al., 2002), we first used path analyses test whether caregiver stress and child emotional distress successively mediated the contribution of COVID-19 economic hardships to children's self-regulation abilities. To control for family income, child gender, and children's self-regulation at T1, we included direct paths from those variables to significant covariances. We used the bootstrap resampling method with 1000 iterations to estimate standard errors and test mediational effects (Hayes, 2013).

**Table 1:** Bivariate correlations among averaged variables.

	**1**.	**2**.	**3**.	**4**.	**5**.	**6**.
1. T1 Income						
2. T1 Child self-regulation	−0.05					
3. T1 COVID-19 economic hardships	−0.41^***^	−0.03				
4. T2 Caregiver stress	−0.14	−0.16	0.34^***^			
5. T2 Child emotional distress	−0.16	−0.10	0.19	0.45^***^		
6. T2 Child self-regulation	0.04	0.70^***^	−0.12	−0.34^***^	−0.34^***^	
7. Child age in months	−0.08	−0.02	−0.12	0.10	0.05	-0.07

*T1, Time 1 survey; T2, Time 2 survey*.

*** *p ≤ 0.001*.

**Figure 1:**
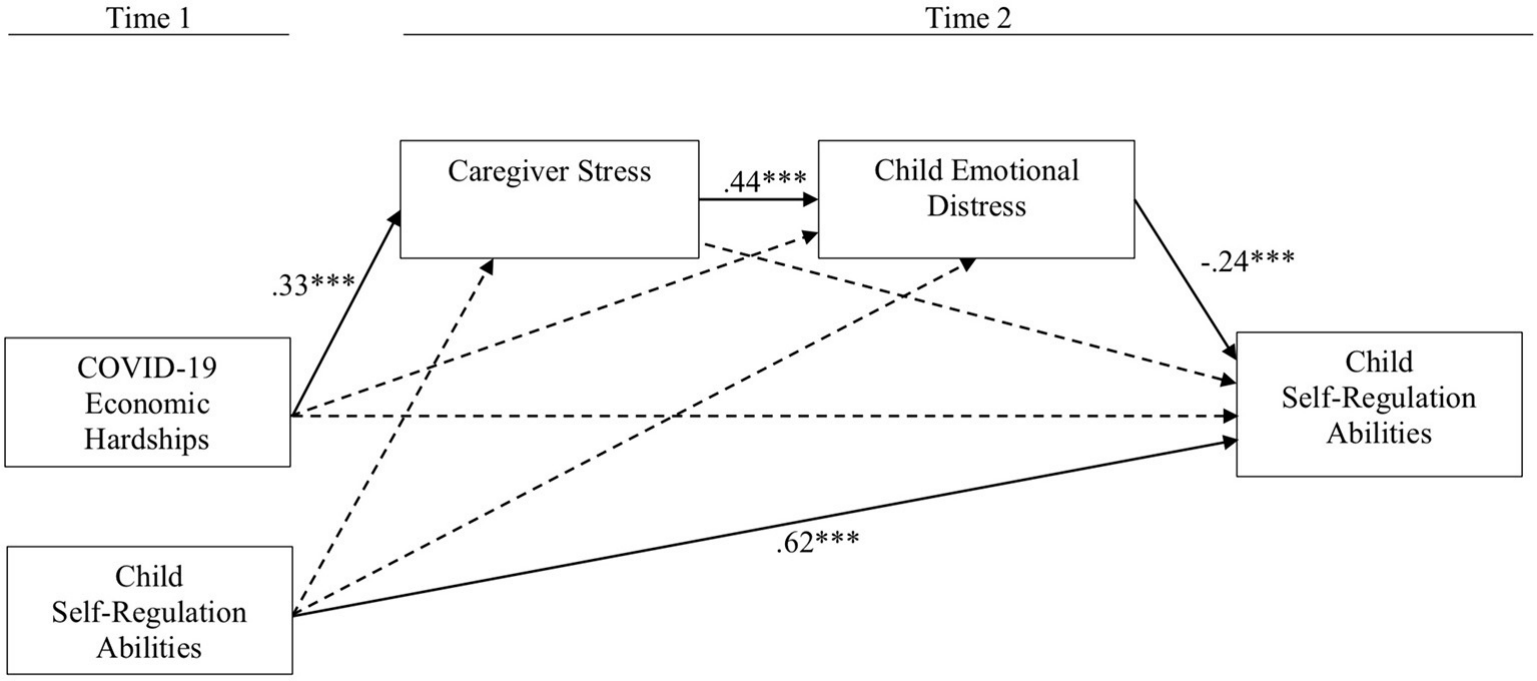
Path model. We controlled for family income and child gender, but these effects were non-significant and omitted from the figure to facilitate interpretation. Dashed paths represent non-significant effects. Standardized estimates shown. χ^2^(3) = 0.58, *p* = 0.90, RMSEA = 0.00, CFI = 1.00. Non-significant chi-squares indicate good model fit, as do RMSEA values <0.05 and CFI >0.90 (Hu and Bentler, 1999). ***p ≤ 0.001.

Our path model fit the data well (Figure 1). The results revealed a negative, indirect association between COVID-19 economic hardships and children's T2 self-regulation. Formal tests of mediation revealed that caregiver stress and child emotional distress successively mediated the association between COVID-19 economic hardships and children's T2 self-regulation, *b* = −0.04, *SE* = 0.02, *p* = 0.02, 95% confidence interval (CI): −0.06 to −0.01. In other words, COVID-19 economic hardships were related to increased caregiver stress, which was associated with higher emotional distress and poorer self-regulation in children.

**Figure 2:**
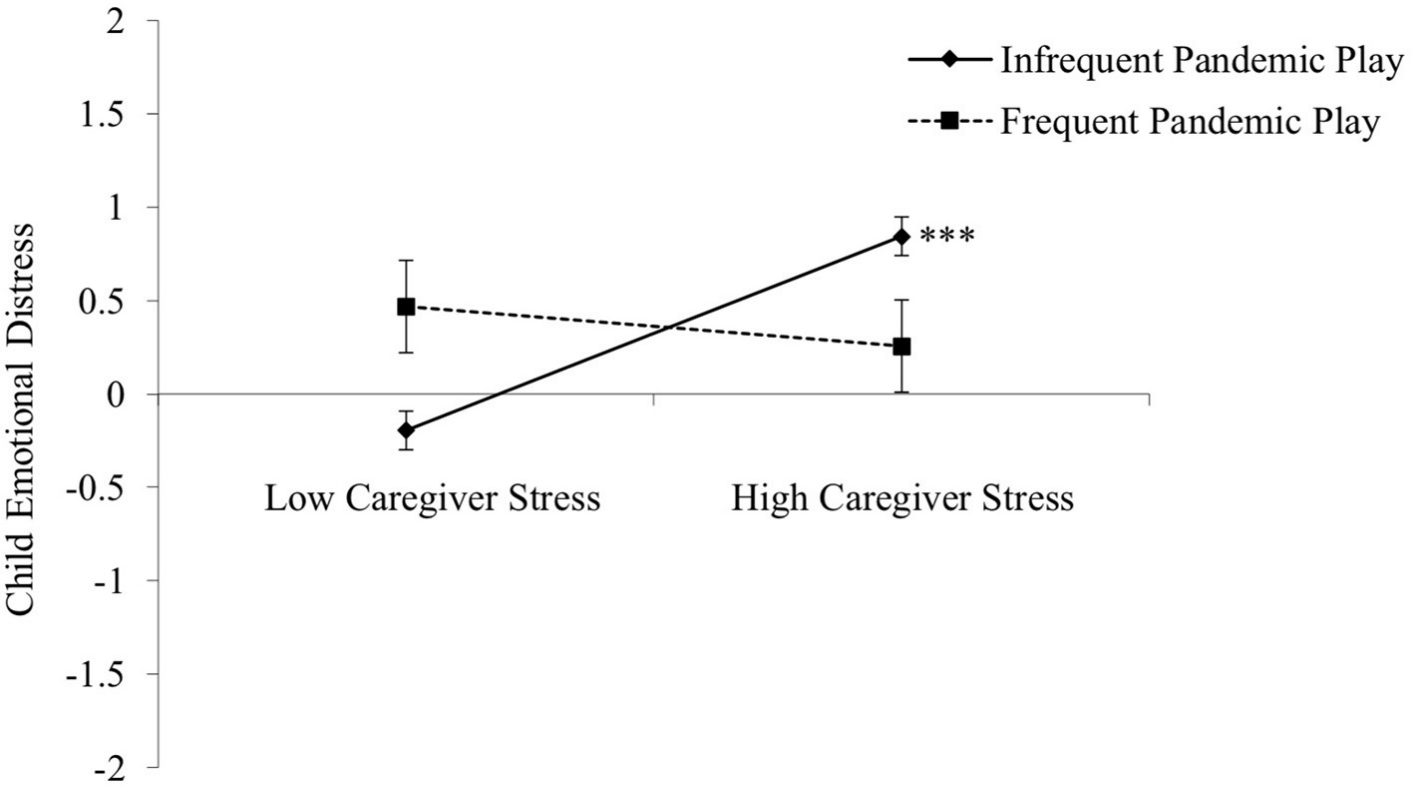
Interaction between caregiver stress and pandemic play on children's emotional distress. High caregiver stress is related to increased emotional distress for children who engaged in pandemic play infrequently, but not for those who engaged in pandemic play frequently. ***p < 0.001.

To examine whether children's engagement in pandemic play moderated the abovementioned associations, we first tested two multi-group path models to compare the pattern of associations between the children who engaged in pandemic play infrequently and those who engaged in it frequently. The pattern of associations was similar across both groups of children, except for two paths: caregiver stress to child emotional distress and child emotional distress to T2 self-regulation. In both cases, the paths were significant for the children who engaged in pandemic play infrequently but non-significant for those who engaged in pandemic play frequently. We followed-up these multi-group path models with more robust moderation analyses that compared the two pandemic play groups to one another rather than examining differences between these groups in separate models. These follow-up analyses revealed the relationship (i.e., simple slopes) between caregiver stress and child emotional distress varied by children's engagement in pandemic play (*b* = −0.71, *SE b* = 0.27, *p* = 0.01, CI: −1.24 to −0.18; Table 2; Figure 2). By contrast, the slopes for the association between children's T2 emotional distress and self-regulation did not vary between the two groups of children, indicating that the frequency of pandemic play did not moderate that association (*b* = 0.15, *SE b* = 0.23, *p* = 0.51, CI: −0.31 to 0.62; see Appendix C in Supplementary Material for full regression statistics). Thus, children's engagement in pandemic play may protect children by mitigating the adverse contribution of caregiver stress to child emotional distress.

**Table 2:** Regression analysis examining how the association between caregiver stress and child emotional distress varied as a function of children's engagement in pandemic play.

**Variable**	***b***	***SE b***	***p***	**95% CI**
Constant	0.32	0.27	0.23	−0.21 to 0.86
Family income	−0.02	0.02	0.36	−0.07 to 0.02
Child gender	−0.23	0.17	0.18	−0.56 to 0.11
Caregiver stress	0.59	0.10	<0.001	0.39 to 0.80
Pandemic play	0.04	0.20	0.85	−0.37 to 0.44
Caregiver stress × Pandemic play	−0.71	0.27	0.01	−1.24 to −0.18

*For Pandemic Play, a score of 0 indicated infrequent Pandemic Play (i.e., never to less than once a week) and a 1 indicated frequent Pandemic Play (i.e., once a week or more). The Caregiver Stress × Pandemic Play variable is the product of Caregiver Stress and Pandemic Play scores*.

Although we relied on theory and research to support the primary path model, we explored the alternative possibility that T2 caregiver stress and children's self-regulation abilities successively mediate the association between T1 COVID-19 economic hardships and T2 children's emotional distress. The alternative path model fit the data well [χ^2^(3) = 0.92, *p* = 0.82, RMSEA = 0.01, CFI = 1.00] and the mediation effect was significant (*b* = 0.03, *SE b* = 0.02, *p* = 0.05, CI: 0.01–0.06). The findings from the primary and alternative path models suggest that there may be a transactional association between children's emotional distress and self-regulation. The correlational nature of these variables precludes a direct test of this hypothesis. Pandemic play moderation analyses mirrored the findings above. Together, our primary and alternative path models suggest that COVID-19 economic hardships heightened children's emotional distress and obstructed their self-regulation abilities by exacerbating caregiver stress. However, engaging in pandemic-related play may reduce children's emotional distress and protect their self-regulation abilities by weakening the extent to which caregiver stress compromises children's emotional adjustment.

## Discussion

The purpose of the present study was to examine the longitudinal processes by which COVID-19 economic hardships contribute to children's emotional distress and self-regulation development and to assess whether pandemic play can serve as a protective factor. As hypothesized, COVID-19 hardships were related to increased caregiver stress, which was associated with increased emotional distress and poorer self-regulation abilities among children. These findings are consistent with prior research on family stress, risk, and resilience (Masarik and Conger, 2017) as well as recent theoretical frameworks for how COVID-19 hardships may impact families with children (Prime et al., 2020; Spinelli et al., 2020).

Our findings also suggest that engaging in pandemic-related play themes may protect young children from the harmful effects of COVID-19 economic hardships by reducing the adverse contribution of caregiver stress on children's emotional distress. Caregiver stress did not appear to heighten the emotional distress of the children who engaged in pandemic play frequently. To our knowledge, this is the first study providing important empirical support to the idea that pandemic-related play may protect children from COVID-19 stressors popularized by numerous media outlets during the pandemic. The idea that play may be an effective means for children to cope with stressful experiences is, however, not new. Play therapy, for example, has been shown to help children cope with trauma and unexpected hardships (e.g., Lin and Bratton, 2015). Yet, play therapy is typically administered in controlled settings with trained therapists guiding children through the play and the processing of their experiences. By contrast, 90% of the children in our sample were reported to initiate pandemic play on their own. As such, this research demonstrates that spontaneous pretend play about unexpected hardships, like the COVID-19 pandemic, could also be an important and accessible way for children cope with stressful experiences.

Previous research and theory on pretend play suggest the following possibilities as to how pandemic play might serve as a protective factor. (1) Engaging in pretend play is typically a fun and enjoyable experience. As children consistently engage in pretend play, they may cultivate a positive environment that could compensate for an otherwise stressful context at home (Russ, 2007; Thibodeau-Nielsen et al., 2020). (2) Pretend play often provides opportunities for caregivers to engage in meaningful and harmonious interactions with their children. In fact, 70% of those reported to engage in pandemic play in our sample did so with an adult at least some of the time. These coordinated interactions between a caregiver and child may help to regulate the child's bodily response to stress (Yogman et al., 2018). (3) Pretend play may provide a safe context in which children can process a wide range of emotions and experiences. Scholars have suggested that reenacting specific stressful events in play is cathartic for children (Russ, 2004). Just as adults verbally share their anxieties or fears as a way to cope with stress, children often express their feelings and thoughts through play (e.g., Chethik, 1989). Doing so may allow any anxieties or fears to gradually fade over time (Knell, 1993; Russ, 2004, 2007). Play may also allow children to assert control over the outcomes of stressful experiences, thus gaining mastery over the situation (Erikson, 1963).

These findings have important implications, even beyond the COVID-19 health crisis. For example, one of the main findings of this study is that COVID-19 economic hardships adversely contributed to children's emotional distress and self-regulation indirectly through caregiver stress. Thus, interventions aimed to reduce caregiver stress levels could play a key role in disrupting the pathway through which family hardships, both during and after the COVID-19 pandemic, can threaten children's well-being. Furthermore, our data suggest that providing children with ample opportunities to engage in pretend play may be an effective way for caregivers to support their children's emotional well-being during difficult times. Prior to the COVID-19 pandemic, the American Academy of Pediatrics published a report urging pediatricians to write prescriptions for play given the associated developmental benefits (Yogman et al., 2018). In line with their suggestion that play may serve as a possible antidote to the negative consequences of adversity, our findings suggest that pretend play may be an especially important tool to promote well-being during the current global health crisis. It is worth reemphasizing that most children in our study were reported to initiate pandemic play on their own; before we can make specific recommendations about caregiver-initiated pandemic play or other interventions involving pandemic play, more research is needed to understand how, for whom, and in what contexts pandemic play benefits well-being. In the meantime, given the developmental benefits associated with pretend play in general (e.g., Thibodeau et al., 2016; White and Carlson, 2016) and its potential to be a protective factor against adversity for diverse populations of children (Yogman et al., 2018; Thibodeau-Nielsen et al., 2020), caregivers could consider creating opportunities for children to engage in pretend play. Doing so may provide a context for child-initiated pandemic play to naturally emerge among children who are ready to process pandemic-related stressors in this way. If a child naturally incorporates pandemic-related themes into their play, caregivers may consider following their child's lead in this play or simply allowing this theme to play out, as our findings suggest it might help some children further adapt to pandemic life. Outside of the COVID-19 pandemic, caregivers may notice their child playing about other stressful or new experiences, like natural disasters or making new friends on the first day of school (e.g., Buchanan et al., 2009). Together, theory and the results of the present study suggest that allowing children to engage in these forms of play as they occur naturally might be an effective way to alleviate emotional distress that stems from other stressful experiences for some children.

This study also has noteworthy limitations. First, all data were gathered from caregiver surveys and thus reflect caregivers' perceptions. Our results may be affected by shared method variance, which can inflate the variable associations. Second, our relatively small sample consisted of mostly White, highly educated, and high-income families. Yet, the experience of economic hardship is subjective and can lead to increased stress regardless of social class (Masarik and Conger, 2017). Still, the extent to which our findings can be generalized to a larger sample of families from other ethnicities, educational backgrounds, and income levels may be limited. Relatedly, the majority of respondents were mothers, limiting conclusions that can be made with respect to other caregivers like fathers or grandparents. Third, we only examined the frequency with which pandemic play occurred. Our data prevented us from examining the contribution of factors like thematic content of the pandemic play, when and where it occurred, and to what extent others were involved. This limits the recommendations we can offer about caregivers' roles in initiating hardship-related play. Future studies of children's play behaviors should gather data from multiple sources in socioeconomically and ethnically diverse samples.

In conclusion, the present study highlights the process by which economic hardships during the COVID-19 pandemic may hinder children's emotional well-being and self-regulation development, with caregiver stress as a key intervening mechanism. Our findings also add to the growing number of studies suggesting that pretend play can protect children from the harmful effects of adversity (e.g., Thibodeau-Nielsen et al., 2020). Although this study was conducted in the context of the COVID-19 pandemic, the findings are likely relevant beyond this particular global health crisis. Many families experience economic hardship every day (Schiller, 2008). Addressing caregiver stress levels and allowing children an outlet to cope with challenges through pretend play could have crucial protective effects on early development and well-being during times of crisis.

## Data Availability Statement

Deidentified data supporting the conclusions of this article will be made available by the authors upon request.

## Ethics Statement

This study involving human participants was reviewed and approved by University of Missouri Institutional Review Board. The participants provided informed consent to participate in this study.

## Author Contributions

RT-N and FP conceptualized and designed the study, collected data, conducted analyses, drafted the initial manuscript, and reviewed and revised the manuscript. RW conceptualized and designed the study, collected data, assisted in interpretation of the findings, and reviewed and revised the manuscript. AW collected data, assisted in data analysis and interpretation, drafted the initial manuscript, and reviewed and revised the manuscript. SD collected data, assisted in interpretation of findings, and reviewed and revised the manuscript. All authors approved the final manuscript as submitted and agree to be accountable for all aspects of the work.

## Conflict of Interest

The authors declare that the research was conducted in the absence of any commercial or financial relationships that could be construed as a potential conflict of interest.

## Publisher's Note

All claims expressed in this article are solely those of the authors and do not necessarily represent those of their affiliated organizations, or those of the publisher, the editors and the reviewers. Any product that may be evaluated in this article, or claim that may be made by its manufacturer, is not guaranteed or endorsed by the publisher.
