# Supplementary material for: Child Adjustment During COVID-19: The Role of Economic Hardship, Caregiver Stress, and Pandemic Play
Source: Front Psychol. 2021 Aug 18;12:716651. doi: 10.3389/fpsyg.2021.716651 (PMC8416273; doi:10.3389/fpsyg.2021.716651)
Supplement: Supplementary file 1 [file Table_1.DOCX]

**Supplementary Appendix A**

Qualitative Examples of COVID-19 Themes in Children’s Play

| “When she was playing with Barbies, the baby asked, ‘Mommy can we go to the playground?’ And the mommy said, ‘No the playground is closed because of the virus.’ ” | “He has pretended to be working from home. He pretends to be on zoom conference calls and like he is teaching a class while on a pretend computer or he sits at ours’ to play.” |
| --- | --- |
| “He has conversations with his toys about school being closed, about feeling sad, and about wearing masks/distancing.” | “On his fake phone calls: ‘Grandma & Grandpa? Yeah, we can't come over because of the virus.’" |
| “Bad guys now spread coronavirus as a weapon.” | “During her veterinarian play, she brought all the cats in for COVID testing.” |
| “[He] has pretend friends that were friends at his preschool that no longer go there because of COVID.” | “They have a play kitchen and will put a chair up to the entrance of the kitchen ‘to keep germs away’.” |
| “Throwing everyone she knows and all her stuffed animals a pretend quarantine birthday theme - she had a quarantine party. She pretend bakes a cake and sets all the animals up on one side of the room for a video call while the birthday animal eats their cake alone.” | “When we're playing with her babies she will be the doctor and talk about how her baby has "the big cold" which is what we call Covid 19 around her. She'll examine the baby and make it better. She'll also put her masks on her stuffed animals when we play sometimes.” |
| “My kids is playing doctor all the time, much more than before. He is consistently pretending to give shots and take the temperatures of his siblings, at least two/three times a week.” | “He is really into magic and fantasy play, and recently he talked about putting a force field around our house to protect us from the virus. He also put one around his Grammy's house, and then around the whole world.” |
| “She has an imaginary brother named Dexter, and she has expressed concern that he would get coronavirus at his daycare (which happens at the stop sign near our house).” | “She has pretended to go to a grocery store. She pretends to put on a mask while she shops with her stuffed animal friends. All of her ‘friends’ have pretend masks on too.” |

**Supplementary Appendix B**

Bivariate Correlations, Means, Standard Deviations, and Ranges Among the Individual Measures

|  | 1. | 2. | 3. | 4. | 5. | 6. | 7. | 8. | 9. | 10. | 11. |
| --- | --- | --- | --- | --- | --- | --- | --- | --- | --- | --- | --- |
| 1. T1 Income |  |  |  |  |  |  |  |  |  |  |  |
| 2. T1 Child cognitive self-regulation | -.11 |  |  |  |  |  |  |  |  |  |  |
| 3. T1 Child behavioral self-regulation | .02 | .39*** |  |  |  |  |  |  |  |  |  |
| 4. T1 Number of economic changes | -.22* | -.02 | -.06 |  |  |  |  |  |  |  |  |
| 5. T1 Financial strain | -.50*** | .11 | -.12 | .53*** |  |  |  |  |  |  |  |
| 6. T2 Parent perceived stress | -.24* | .04 | -.10 | .23* | .24* |  |  |  |  |  |  |
| 7. T2 Parenting stress | -.01 | -.18 | -.22* | .34*** | .23* | .54*** |  |  |  |  |  |
| 8. T2 Child anxiety/depression | -.26** | -.14 | -.04 | .18 | .19 | .38*** | .39*** |  |  |  |  |
| 9. T2 Child emotional reactivity | -.05 | -.10 | -.04 | .17 | .08 | .35*** | .39*** | .79*** |  |  |  |
| 10. T2 Child cognitive self-regulation | -.07 | .60*** | .48*** | -.06 | .10 | -.07 | -.25* | -.17 | -.14 |  |  |
| 11. T2 Child behavioral self-regulation | .14 | .30** | .64*** | .21* | -.19 | -.33*** | -.38*** | -.39*** | -.42*** | .51*** |  |
| *M* | 11.41 | 3.62 | 3.74 | 1.00 | 1.75 | 2.80 | 2.77 | 2.14 | 3.00 | 3.72 | 3.80 |
| *SD* | 3.76 | .59 | .65 | 1.07 | .91 | .64 | .67 | 2.19 | 2.95 | .60 | .74 |
| *Range* | 1–15 | 2–5 | 2–5 | 0–4 | 1–4 | 1.20–4.30 | 1.10-4 | 0–11 | 0–13 | 2.2–5 | 2–5 |

*Note.* T1 = Time 1 survey; T2 = Time 2 survey. **p* < .05, ***p* < .01, ****p* < .001.

**Supplementary Appendix C**

Regression Analysis Examining how the Association Between Children’s Emotional Distress and Self-Regulation at Time 2 Varies as a Function of Children’s Engagement in Pandemic Play

| Variable | *b* | *SE b* | *p* | 95% CI |
| --- | --- | --- | --- | --- |
| Constant | .23 | .26 | .39 | -.29 to .74 |
| Family Income | .01 | .02 | .70 | -.03 to .05 |
| Child Gender | -.57 | .16 | .001 | -.89 to -.26 |
| Child Emotional Distress | -.36 | .09 | < .001 | -.55 to -.18 |
| Pandemic Play | -.19 | .19 | .32 | -.58 to .19 |
| Child Emotional Distress × Pandemic Play | .15 | .23 | .51 | -.31 to .62 |

*Note:* For Pandemic Play a score of 0 indicated infrequent Pandemic Play (i.e., never to less than once a week) and a 1 indicated frequent Pandemic Play (i.e., once a week or more). The Child Emotional Distress × Pandemic Play variable is the product of Child Emotional Distress and Pandemic Play scores.
